# Phonon-assisted nearly pure spin current in DNA molecular chains: a multifractal analysis

**DOI:** 10.1038/s41598-023-48644-x

**Published:** 2023-12-02

**Authors:** S. Fathizadeh

**Affiliations:** 1 Department of Physics, Urmia University of Technology, Urmia, Iran; 2 Research Institute for Applied Physics and Astronomy, Tabriz University, Tabriz, Iran

**Keywords:** Biophysics, Materials science, Physics

## Abstract

Motivated by the development of molecular spintronics, we studied the phonon-assisted spin transport along a DNA chain in the presence of environmental-induced dephasing using multifractal analysis. The results demonstrate that a nearly pure spin current is generated in the presence of the voltage gate. The pure spin current is enhanced by increasing thermal effects. The vibration modes due to the thermal phonon bath assist in generating the spin current, so the spin state is more delocalized in strong electron-phonon coupling. The phonon chirality can translate to the electron spin to create a nontrivial spin texture, including spin currents. The spin states become more extended by increasing the phonon temperature. On the other hand, the spin states are less localized in longer chains as the spin selectivity is higher in longer chains than in short ones. Therefore, we can engineer a molecular spintronic device by controlling phonon effects on the storage and transport of binary digits.

## Introduction

Spintronics uses the spin degree of freedom of electrons, rather than their charge, for potential quantum sensing and quantum measurement applications. The critical components of spintronics are the creation, injection, transfer, measurement, and control of spin currents, including the pure spin current and the spin-polarized charge current^1,2^. The pure spin current helps achieve spin accumulation and spin transport in nanodevices due to its ability to modify the spin state in the device region with no significant charge transfer^3^. In low-dimensional structures, spin-orbit (SO) interaction, especially the Rashba SO interaction, induces an electric potential that could break the space inversion symmetry^4,5^. Then, it is possible to manipulate the spin state by electrical stimulation. The use of organic materials as electronic components, rather than conventional silicon-based semiconductors, is a topic of intense interest for researchers and manufacturers. Organic materials offer potential benefits for spintronics, as they have specific properties that differ from conventional materials^6–8^. Organic materials have low costs, high chemical reactivity, mechanical adaptability, and simple fabrication methods^7^. Molecular spintronics appeared from the investigation of possible uses of molecules or the improvement of existing spintronics devices with novel organic materials. DNA, an organic material, has various functional and unique features, including self-organization, long-distance electron movements, the possibility of creating artificial chiral systems with DNA, spin-dependent transport, and spin polarization that make it suitable for spintronics applications in nano scales^9^. The models incorporating the electron-phonon coupling to describe the spin selectivity in chiral molecules can offer more insight into the chiral-induced spin selectivity (CISS) effect than simplified models^10,11^. The chiral structure undergoes spin polarization in response to the charge transport. The charge transport dynamics reveals that CISS is a phenomenon of the excited state that, in the transient regime, can be partly accounted for by a simple single-particle description. In the steady-state limit, electron correlations, such as electron-vibration interactions, are critical in preserving an inherent spin anisotropy that can lead to nonvanishing spin selectivity^12^.

Recently, several model computations have resulted in large spin polarization considering polaron charge transport^10^ or phonon resonance effects^11,13^. Moreover, the reports suggest that the CISS mechanism must include some extra functions, as a correlation was observed between the degree of spin polarizations, chiral molecules, and their optical activity^14,15^. Recent theoretical advances highlight the significant role of electronic interactions and indicate the need to consider CISS from an excited state perspective^10,11,13,16–18^. The concept of excited states encompasses virtual excitations, electron dispersions induced by temporal fluctuations, and vibrational excitations of molecules that couple with electrons and alter their electronic structures. Various interactions, including Coulomb^19^ and electron-vibration interactions^20^, polarons^10^, photoexcitations^16^, time dependence^17^, and dissipation^21^, result in CISS measurable effects.

Spin-phonon coupling influences electron transport properties since it is used in spin mechanics setups for quantum science and technology. Then, the ability to manipulate them is of particular interest^22^. Spin-phonon interaction is essential because phonons can drive spin-lattice relaxation, which results in a reduction of coherence time, one of the main concerns for quantum computing^23^.

In this regard, we have chosen the DNA chain as a chiral biological molecule to study its spin transport properties in the presence of phonon effect. We have considered the general Hamiltonian to describe the spin-phonon interaction and its influence on spin currents in DNA molecules. Multifractal analysis is a standard method in studying localization-delocalization transition^24^. We use the multifractal analysis to reveal the critical behavior of the system. Using the multifractal analysis, we can obtain novel results and verify the earlier experimental findings. The electron-phonon coupling and thermal effects on the spin transport of DNA are theoretically examined. Therefore, we can find the range of control parameters for the localization/delocalization of a spin current. Such localized/delocalized spin currents can be used for designing an optoelectrical device based on DNA biomolecules to save, transport, and process information.

## Model

We model a DNA chain sandwiched between left and right metal leads and indicate all relevant structural parameters. Following Hamiltonian can capture the spin transport:

1 $$\begin{aligned} {\mathscr {H}}={\mathscr {H}}_{chain}+{\mathscr {H}}_{lead}+{\mathscr {H}}_{chain-lead}+{\mathscr {H}}_{d} \end{aligned}$$

${\mathscr {H}}_{chain}$ is the Hamiltonian of the DNA molecular chain and includes the following terms:

2 $$\begin{aligned} {\mathscr {H}}_{chain}=H_{e}+H_{so}+H_{ph}+H_{e-ph} \end{aligned}$$

where, $H_{e}$ is a Hamiltonian term that accounts for the electron transport in an *N*-base pair DNA ladder model, including the spin degree of freedom as follows^25,26^:

3 $$\begin{aligned} H_{e}=\sum _{n}\left[ \sum _{m=1,2} \left( \varepsilon _{n,m}c_{n,m}^{\dagger }c_{n,m} +t_{n,n+1}^{(m)}c_{n,m}^{\dagger }c_{n+1,m}\right) +\lambda c_{n,1}^{\dagger }c_{n,2}+H.c.\right] \end{aligned}$$

where, $c_{n,m}^{\dagger }=\left( c_{n,m,\uparrow }^{\dagger },c_{n,m,\downarrow }^{\dagger }\right)$ and $c_{n,m}=\left( c_{n,m,\uparrow },c_{n,m,\downarrow }\right) ^T$ are the creation and annihilation operators of a spinor in site *n* of strand *m* of DNA. $\epsilon _{n,m}$ is the on-site energy of an electron in site (*n*, *m*) chosen as $\epsilon _{n,1}=0$ and $\epsilon _{n,2}=0.3~\textrm{eV}$. $t_{n,n+1}^{(m)}$ and $\lambda$ are the longitudinal and transverse hopping constants between the neighboring bases, respectively, are taken as $t_{n,n+1}^{(2)}=0.1~\textrm{eV}$, $\lambda =-0.08~\textrm{eV}$ and $t_{n,n+1}^{(1)}=-xt_{n,n+1}^{(2)}$, where $x=1.4$ is an additional parameter to describe the asymmetry between two helicoidal chains^11,27^. When the DNA molecule is subjected to a perpendicular electric field, the on-site energy of site (*n*, *m*) is corrected as $\varepsilon _{n,m}=\varepsilon _{n,m}^{0}-(-1)^{m}eV_{g}\cos [(n-1)\Delta \varphi ]$, where $\varepsilon _{n,m}^{0}$ is the on-site energy in the absence of gating voltage ($V_{g}=E_{g}2R$). Here, $E_{g}$ is the perpendicular electrical field, and 2*R* is the effective distance between the complementary bases.

$H_{so}$ demonstrates a Rashba-like spin-orbit coupling (SOC) generated through the inversion symmetry breaking due to creating an internal field in the DNA double strand. When an electron with mass $m_{e}$, charge *e*, and momentum operator $\hat{\vec {P}}$ moves in the electrostatic potential *V*, the SOC arises as $H_{so}=\frac{\hbar }{4m_{e}^{2}c^{2}}\nabla V.({\hat{\sigma }}\times \hat{\vec {P}})$, ${\hat{\sigma }}=(\sigma _{x},\sigma _{y},\sigma _{z})$ are Pauli matrices^27^. In second quantization representation, $H_{so}$ is written as follows^25^:

4 $$\begin{aligned} H_{so}= & {} \sum _{n}\sum _{m}\left[ it_{so}^{(m)}c_{n,m}^{\dagger }\left( \sigma _{n}^{(m)}+\sigma _{n+1}^{(m)}\right) c_{n+1,m}+H.c.\right] \end{aligned}$$

where, $t_{so}^{(m)}$ is SOC taken as $t_{so}^{(1)}=0.01~\textrm{eV}$ and $t_{so}^{(2)}=x~t_{so}^{(1)}$. The term $\sigma _{n+1}^{(m)}=\sigma _{z}\cos \theta -(-1)^{m}[\sigma _{x}\sin \varphi -\sigma _{y}\cos \varphi ]\sin \theta$, with $\theta =0.66~rad$ as the helix angle, $\varphi =n\Delta \phi$ the cylindrical coordinate, and $\Delta \phi =\pi /5$ the twist angle^27,28^.

$H_{ph}$ and $H_{e-ph}$ characterize the phonon energy with phonon frequency $\omega _{0}$ and interaction between electrons and phonon with site-independent strength $\Lambda$ as follows^29^:

5 $$\begin{aligned} H_{ph}+H_{e-ph}=\sum _{q}\omega _{0}b_{q}^{\dagger }b_{q}+\sum _{n,m}\Lambda (b_{0}^{\dagger }+ b_{0})c_{n,m}^{\dagger }c_{n,m} \end{aligned}$$

where, $\Lambda$ is reduced to $\Lambda _{0}$ for on-site electrons and $M_{1}$ and $M_{2}$, for intrachain and interchain nearest neighbor hopping electrons of the DNA chain, respectively^30^. It is reasonable to suppose $M_{1(2)}< \Lambda _{0}$ in a double-stranded DNA chain. For our calculations, we set $M_{1}=M_{2}=0.2~\Lambda _{0}$^30^. We have connected the central molecular system to the right and left real electrodes with potential difference $V_{b}$ along the chain. ${\mathscr {H}}_{lead}$ and ${\mathscr {H}}_{chain-lead}$ describe the Hamiltonian of leads and interaction of leads with the molecule, respectively, as follows^31,32^:

6 $$\begin{aligned} {\mathscr {H}}_{lead}= & {} \sum _{k,m}\left[ \varepsilon _{L_{k,m}}a_{L_{k,m}}^{\dagger }a_{L_{k,m}} + \varepsilon _{R_{k,m}}a_{R_{k,m}}^{\dagger }a_{R_{k,m}}\right] \end{aligned}$$

7 $$\begin{aligned} {\mathscr {H}}_{chain-lead}= & {} \sum _{k,m}\left[ t_{L}a_{L_{k,m}}^{\dagger }c_{1,m} +t_{R}a_{R_{k,m}}^{\dagger }c_{N,m}+H.c.\right] \end{aligned}$$

where $a_{\beta _{k,m}}^{\dagger } (a_{\beta _{k,m}})$ and $\beta =L,R$ is the creation (annihilation) operator in site *k* of strand *m* in lead $\beta$, $\varepsilon _{\beta _{k,m}}$ is the on-site energy of leads, and $t_{\beta }$ is the hopping energy between the leads and central DNA molecule^33^.

**Figure 1:**
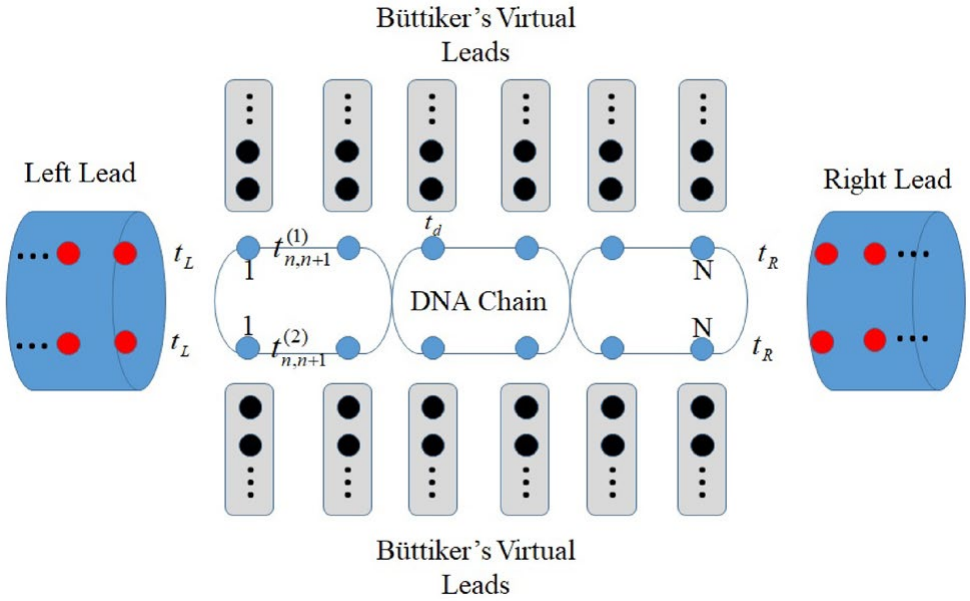
A schematic illustration of a DNA chain connected to the left and right leads and immersed in a vibrational phonon bath. Each base of the DNA chain is connected to one Büttiker’s virtual lead. The blue circle on the DNA chain shows DNA bases. The red and black circles are presented as the sites of metal leads and virtual leads, respectively. All hopping parameters and couplings between DNA and leads are shown.

To describe the dephasing processes caused by the electrons’ inelastic scattering with the electrons, the phonons, the counterions, and the adsorbed impurities, we introduce the dephasing term ${\mathscr {H}}_{d}$ as follows^27,34^:

8 $$\begin{aligned} {\mathscr {H}}_{d}= & {} \sum _{n,m,k}\left[ \varepsilon _{n,m,k}d_{n,m,k}^{\dagger }d_{n,m,k}+t_{d}d_{n,m,k}^{\dagger }c_{n,m}+H.c.\right] \end{aligned}$$

where the phase-breaking processes can be modeled by connecting each site of the DNA molecule to a Büttiker’s virtual lead with $d_{n,m,k}^{\dagger }$ as the creation operator of mode *k* and $t_{d}$ as the coupling between the DNA molecule and the virtual lead. A simple schematic illustration of the model is presented in Fig. 1.

The strong electron-phonon coupling leads to the small polaron transport. To describe the phonon effects, we use Lang-Firsov unitary transformation as $\widetilde{{\mathscr {H}}}=e^{-V}{\mathscr {H}}e^{V}$ with $V=\frac{\Lambda _{0}}{\omega _{0}}\sum _{n,m}c_{n,m}^{\dagger }c_{n,m}(b_{0}^{\dagger }-b_{0})+ \sum _{n,m}\left( \frac{M_{1}}{\omega _{0}}c_{n,m}^{\dagger }c_{n+1,m}+\frac{M_{2}}{\omega _{0}}c_{n,1}^{\dagger }c_{n,2})(b_{0}^{\dagger }-b_{0}\right)$^35^. Therefore, the Hamiltonian is transformed to the following form^36,37^:

9 $$\begin{aligned} \widetilde{{\mathscr {H}}}=\widetilde{{\mathscr {H}}}_{chain}+\widetilde{{\mathscr {H}}}_{lead} +\widetilde{{\mathscr {H}}}_{chain-lead}+\widetilde{{\mathscr {H}}}_{d} \end{aligned}$$

and

10 $$\begin{aligned} \widetilde{{\mathscr {H}}}_{chain}={\widetilde{H}}_{e}+{\widetilde{H}}_{so} \end{aligned}$$

where, in the transformed Hamiltonian ${\widetilde{\varepsilon }}_{n,m}=\varepsilon _{n,m}-\frac{\Lambda _{0}^{2}}{\omega _{0}}$, ${\widetilde{t}}_{n,n+1}=t_{n,n+1}-\frac{2\Lambda _{0} M_{1}}{\omega _{0}}$, ${\widetilde{\lambda }}=\lambda -\frac{\Lambda _{0} M_{2}}{\omega _{0}}$, ${\widetilde{t}}_{so}=t_{so}-\frac{2\Lambda _{0} M_{1}}{\omega _{0}}$, ${\widetilde{\varepsilon }}_{\beta _{k,m}}=\varepsilon _{\beta _{k,m}}-\frac{\Lambda _{0}^{2}}{\omega _{0}}$, ${\widetilde{\varepsilon }}_{n,m,k}=\varepsilon _{n,m,k}-\frac{\Lambda _{0}^{2}}{\omega _{0}}$, and ${\widetilde{t}}_{L(R,d)}=t_{L(R,d)}\exp \left[ -\left( \frac{\Lambda _{0}}{\omega _{0}}\right) ^{2}\left( N_{0}+\frac{1}{2}\right) \right]$. Here, $N_{0}=\frac{1}{e^{\frac{\omega _{0}}{KT}}-1}$ is the equilibrium Boson distribution with the temperature *T*.

## Results

Studies on the CISS effect require a measurable quantity sensitive to the electron’s spin. Spin-dependent currents are the proper quantities to monitor the spin selectivity in the system.

### Spin-dependent currents

11 $$\begin{aligned} {\hat{I}}^{\uparrow }(t)= & {} \sum _{n}\bigg \{\sum _{m}\bigg [({\widetilde{t}}_{n,n+1} +2i{\widetilde{t}}_{SO}\cos \theta )c_{n+1,m,\uparrow }^{\dagger }c_{n,m,\uparrow } -\left( {\widetilde{t}}_{n,n-1}-2i{\widetilde{t}}_{SO}\cos \theta \right) c_{n-1,m,\uparrow }^{\dagger }c_{n,m,\uparrow }\nonumber \\{} & {} -(-1)^{m}D_{n,n+1}c_{n+1,m,\uparrow }^{\dagger }c_{n,m,\downarrow }+(-1)^{m}D_{n-1,n}^{*}c_{n-1,m,\uparrow }^{\dagger }c_{n,m,\downarrow }\nonumber \\{} & {} +{\widetilde{t}}_{L}\sum _{k}\delta _{n,1}\left( c_{n,m,\uparrow }^{\dagger }a_{L_{k,m}}-a_{L_{k,m}}^{\dagger }c_{n,m,\uparrow }\right) -{\widetilde{t}}_{R}\sum _{k}\delta _{n,N}\left( c_{n,m,\uparrow }^{\dagger }a_{R_{k,m}}-a_{R_{k,m}}^{\dagger }c_{n,m,\uparrow }\right) \nonumber \\{} & {} +{\widetilde{t}}_{d}\sum _{k}\left( c_{n,m,\uparrow }^{\dagger }d_{n,m,k}-d_{n,m,k}^{\dagger }c_{n,m,\uparrow }\right) \bigg ] +{\widetilde{\lambda }}\left( c_{n,2,\uparrow }^{\dagger }c_{n,1,\uparrow }-c_{n,1,\uparrow }^{\dagger }c_{n,2,\uparrow }\right) \bigg \} \end{aligned}$$

12 $$\begin{aligned} {\hat{I}}^{\downarrow }(t)= & {} \sum _{n}\bigg \{\sum _{m} \bigg [\left( {\widetilde{t}}_{n,n+1}-2i{\widetilde{t}}_{SO}\cos \theta \right) c_{n+1,m,\downarrow }^{\dagger }c_{n,m,\downarrow } -\left( {\widetilde{t}}_{n,n-1}+2i{\widetilde{t}}_{SO}\cos \theta \right) c_{n-1,m,\downarrow }^{\dagger }c_{n,m,\downarrow }\nonumber \\{} & {} -(-1)^{m}D_{n,n+1}^{*}c_{n+1,m,\downarrow }^{\dagger }c_{n,m,\uparrow } +(-1)^{m}D_{n-1,n}c_{n-1,m,\downarrow }^{\dagger }c_{n,m,\uparrow }\nonumber \\{} & {} +{\widetilde{t}}_{L}\sum _{k}\delta _{n,1}\left( c_{n,m,\downarrow }^{\dagger }a_{L_{k,m}}-a_{L_{k,m}}^{\dagger }c_{n,m,\downarrow }\right) -{\widetilde{t}}_{R}\sum _{k}\delta _{n,N}\left( c_{n,m,\downarrow }^{\dagger }a_{R_{k,m}}-a_{R_{k,m}}^{\dagger }c_{n,m,\downarrow }\right) \nonumber \\{} & {} +{\widetilde{t}}_{d}\sum _{k}\left( c_{n,m,\downarrow }^{\dagger }d_{n,m,k}-d_{n,m,k}^{\dagger }c_{n,m,\downarrow }\right) \bigg ] +{\widetilde{\lambda }}\left( c_{n,2,\downarrow }^{\dagger }c_{n,1,\downarrow }-c_{n,1,\downarrow }^{\dagger }c_{n,2,\downarrow }\right) \bigg \} \end{aligned}$$

where, $$D_{n,n+1}=i{\widetilde{t}}_{SO}\sin \theta (\sin n\phi +\sin (n+1)\phi +i\cos n\phi +i\cos (n+1)\phi )$$.

**Figure 2:**
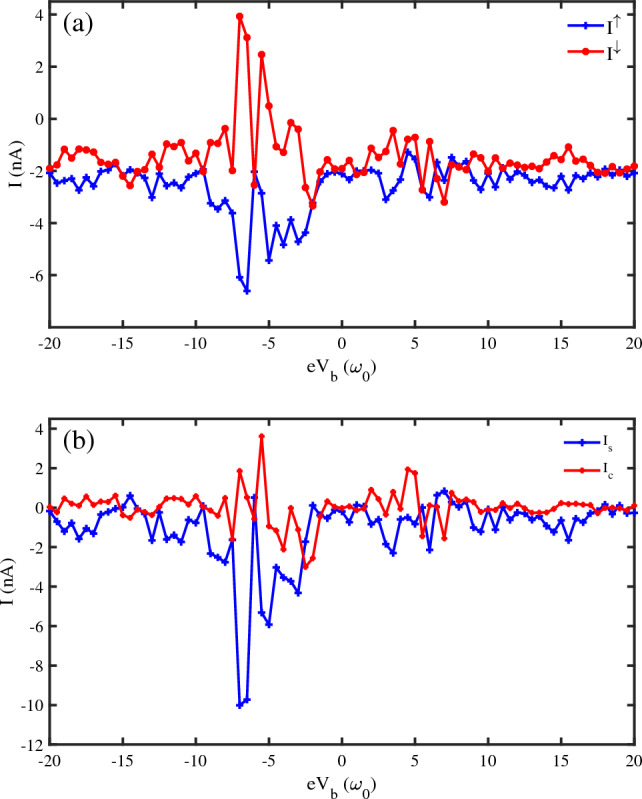
(**a**) The spin-dependent currents $I^{\uparrow }$ and $I^{\downarrow }$ versus bias voltage $(eV_{b})$, (**b**) the net spin current $(I_{s})$ and charge current $(I_{c})$ versus bias voltage $(eV_{b})$, for a $N=50~bp$ DNA chain ($\omega _{0}=0.5~\textrm{eV}$, $eV_g=0.1~\omega _{0}$, $KT=0.1~\omega _{0}$, $\Lambda _{0}=0.1~\omega _{0}$).

We have obtained the spin-dependent currents concerning the bias voltage $(V_{b})$ applied to the DNA chain. Generating the pure spin current is a preferred method to transport information without Ohmic dissipation. The pure spin current with the null charge current can be generated as $eV_b$ changes. Figure 2a displays the spin-up and spin-down currents for different values of bias voltage. We have changed $eV_{b}$ in the range $[-20-20]\omega _{0}$ to observe the spin-up current $(I^{\uparrow })$ and spin-down current $(I^{\downarrow })$ variations. For some voltage values, $I^{\uparrow }$ and $I^{\downarrow }$ flow in opposite directions. The opposite spin-dependent currents are more noticeable for negative voltage values, where the applied voltage direction is reversed. It is observed that DNA can operate as a spin current rectifier roughly.

The other measures for investigation of the spin-dependent currents are the net spin current $(I_{s})$ and the net charge current $(I_{c})$ defined as follows:

13 $$\begin{aligned} I_{s}= & {} I^{\uparrow }-I^{\downarrow }\nonumber \\ I_{c}= & {} I^{\uparrow }+I^{\downarrow } \end{aligned}$$

We have investigated the variation of the net spin and charge currents for different values of voltage bias $eV_{b}$ in Fig. 2b. $I_c$ is diminished almost to zero in a vast region of device voltage settings, implying that a nearly pure spin current is achieved in the device. These properties suggest that we achieve an efficient way to read the corresponding spin states without any energy loss since the charge current is prohibited while the nearly pure spin current is generated. We can report the phenomenon of spin current rectification in a helix, subjected to a transverse electric field, within a tight-binding framework. In the presence of gate voltage $V_{g}$ , associated with the electric field $E_{g} (V_{g} = 2E_{g}R)$, the site energies are modulated through the relation $\varepsilon _{n,m}=\varepsilon _{n,m}^{0}-(-1)^{m}eV_{g}\cos [(n-1)\Delta \varphi ]$. This site energy expression looks similar to the well-known cosine form that is used in the Aunry-André-Harper (AAH) model. $V_{g}$ breaks the symmetry among up and down spin currents and yields different spin-specific junction currents in two bias polarities, resulting in a spin current rectification.

**Figure 3:**
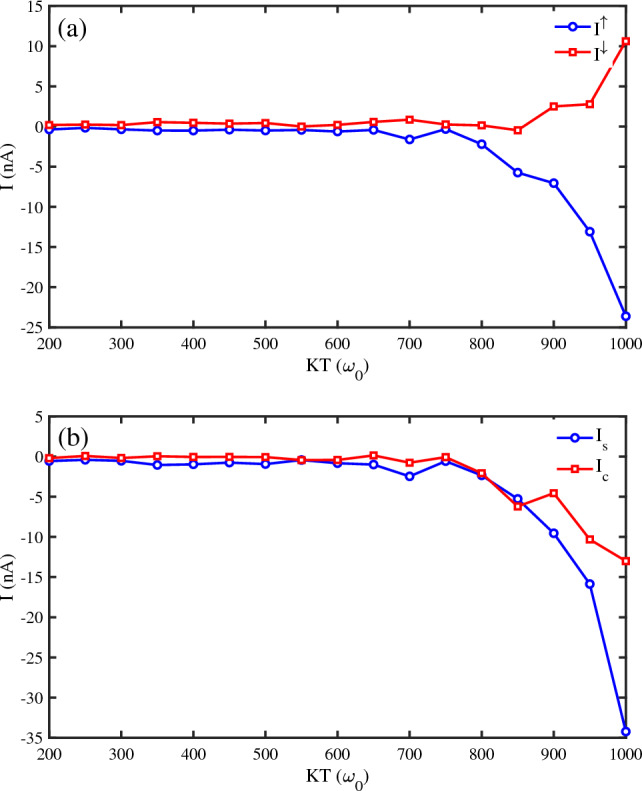
(**a**) The spin-dependent currents $I^{\uparrow }$ and $I^{\downarrow }$ versus phonon temperature (*KT*), (**b**) the net spin current $(I_{s})$ and charge current $(I_{c})$ versus phonon temperature (*KT*), for a $N=50~bp$ DNA chain ($\omega _{0}=0.5~\textrm{eV}$, $eV_g=0.1~\omega _{0}$, $\Lambda _{0}=0.1~\omega _{0}$).

In the following, we have calculated the spin-dependent currents for different values of phonon temperature. The phonon temperature (*KT*) is varied from low temperatures to $1000 \omega _{0}~(\omega _{0}=0.5~\textrm{eV})$ (Fig. 3a,b). It is evident in Fig. 3a that until $KT=700~\omega _{0}$ the spin currents are negligible. Spin currents rise due to increasing temperature. From $KT=800~\omega _{0}$, the spin-up and spin-down currents flow oppositely. Therefore, it is feasible that a nearly pure spin current is generated. To compare the generating of the pure spin current, we obtain the net spin and charge currents in our setup (Fig. 3b). It is observed that until $KT=700~\omega _{0}$, $I_{c}\approx 0$ and $I_{s}$ is low. When *KT* increases, the net spin current reaches higher negative values. From Fig. 3b, one can infer that the dominant spin current belongs to up spins flowing opposite the down spins.

### The effect of gate voltage on Multifractal properties of the system

**Figure 4:**
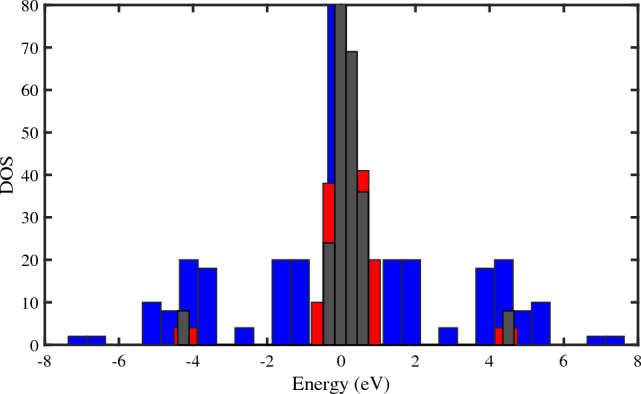
The spin density of states (*DOS*) versus the energy (*E*) for a $N=50~bp$ DNA chain at low voltages ($\omega _{0}=0.5~\textrm{eV}$, $KT=0.1~\omega _{0}$, $\Lambda _{0}=0.1~\omega _{0}$). The blue histogram represents for $eV_g=10~\omega _{0}$, and the red and black histograms are represented for $eV_g=1~\omega _{0}$ and $eV_{g}=0.1~\omega _{0}$, respectively.

In molecular spintronics, it is possible to generate a spin crossover in a molecular nano device exerting voltage. This means that electric current can influence the spin-orbit interaction, which can be adjusted by using gate voltage. Gate voltage can be used to tune the spin-orbit interaction^38,39^. We have explored how gate voltage affects the multifractal features of DNA setup, which can show different localization-delocalization behaviors depending on the gate voltage. We have calculated the density of states (DOS) for different values of applied voltage. Figure 4 shows the DOS for $eV_{g}=0.1~\omega _{0}$ (black color), $eV_{g}=1~\omega _{0}$ (red color), and $eV_{g}=10~\omega _{0}$ (blue color). The figure indicates that the DOS is extended as the gate voltage increases and the spin states become more delocalized. This implies that a transition from localization to delocalization happens when $eV_{g}$ increases.

**Figure 5:**
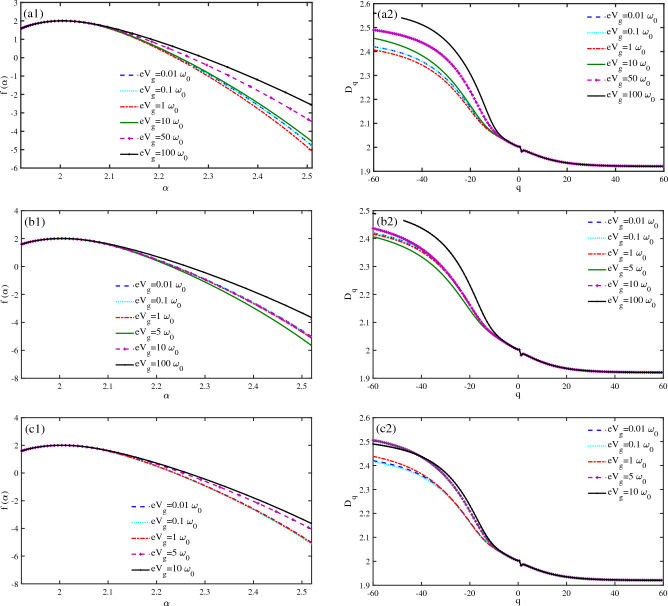
Multifractal analysis of spin states of a $N=50~bp$ DNA chain ($KT=0.1~\omega _{0}$, $\Lambda _{0}=0.1~\omega _{0}$). The singularity spectrum $f(\alpha )$ versus $\alpha$ is presented for different gate voltages $(eV_{g})$ at (**a**1) $\omega _{0}=0.1 eV$, (**b**1) $\omega _{0}=0.05~\textrm{eV}$, and (**c**1) $\omega _{0}=0.5~\textrm{eV}$. The fractal dimensions $D_{q}$ versus *q* are shown for different gate voltages at (**a**2) $\omega _{0}=0.1 eV$, (**b**2) $\omega _{0}=0.05~\textrm{eV}$, and (**c**2) $\omega _{0}=0.5~\textrm{eV}$.

We have used the multifractal analysis to confirm the results for DOS at various voltages. We have studied how the phonon energy of a $N=50~bp$ DNA lattice and the gate voltage affect the multifractal features of the system together. The singularity spectrum $(f(\alpha ))$ and the fractal dimension $(D_{q})$ at $\omega _{0}=0.1~\textrm{eV}$ for various values of $eV_{g}$ are shown in Fig. 5a2. The figure shows that the $f(\alpha )$ width becomes smaller until $eV_{g}=10~\omega _{0}~\textrm{eV}$ (Fig. 5a1). This implies that the spin states become more delocalized as the voltages increase at low voltage ranges. However, when $eV_{g}$ reaches $eV_{g}=10~\omega _{0}~\textrm{eV}$, the voltage effect changes, and the $f(\alpha )$ width becomes larger as the gate voltage increases to high values. The fractal dimension also decreases for negative values of *q* as the gate voltage increases at low values of $eV_{g}$ (Fig. 5a2). However, when the gate voltage reaches $eV_{g}=10~\omega _{0}~\textrm{eV}$, the fractal dimension increases as the gate voltage increases. We have examined $f(\alpha )$ and $D_{q}$ for various values of $eV_{g}$ at $\omega _{0}=0.05~\textrm{eV}$ (Fig. 5b1,b2). The $f(\alpha )$ diagram shows that the spin state becomes more delocalized as the voltage increases until $eV_{g}=10~\omega _{0}~\textrm{eV}$, but after that, the spin state becomes more localized as the gate voltage increases. The results for $D_{q}$ also show that the fractal dimension decreases as the singularity spectrum narrows in low voltages, and the fractal dimension increases as the singularity spectrum becomes wider in higher voltages (Fig. 5b2). We have also studied how the gate voltage affects the multifractal properties of DNA spin states at $\omega _{0}=0.5~\textrm{eV}$ (Figs. 5c1,c2). The $f(\alpha )$ diagram shows that the multifractality increases when the gate voltage reaches $eV_{g}=1~\omega _{0}~\textrm{eV}$ or higher (Fig. 5c1). This means the DNA spin state is more localized at $eV_{g}=5~\omega _{0}~\textrm{eV}$ and higher voltages. The $D_{q}$ diagram shows an increase in fractal dimension at negative *q* values when the gate voltage becomes higher than $eV_{g}=1~\omega _{0}~\textrm{eV}$ (Fig. 5c1). The results indicate a critical value for the gate voltage, below which the multifractality decreases as the voltage increases. In this case, the $f(\alpha )$ becomes narrower, and the spin states are more delocalized. However, above the critical gate voltage, the multifractality increases as the voltage increases, and the spin states become more localized. The delocalized states correspond to lower fractal dimensions and vice versa. As already pointed out, the change of $V_{g}$ regulates the spin-dependent energy spectra, and therefore, by adjusting Fermi energy at suitable places on the band spectra, we can have favorable responses.

### The electron-phonon coupling and multifractal analysis

**Figure 6:**
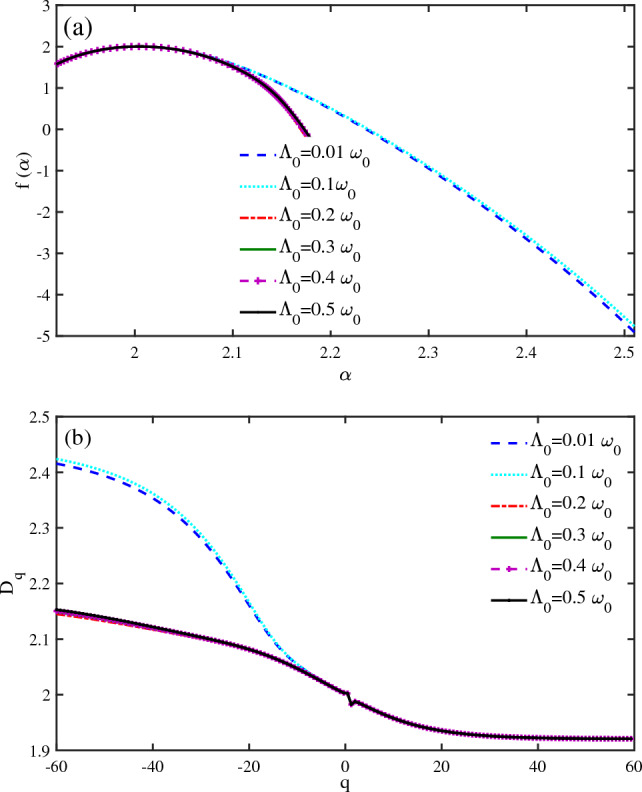
Multifractal analysis of spin states of a $N=50~bp$ DNA chain ($\omega _{0}=0.5~\textrm{eV}$, $KT=0.1~\omega _{0}$, $eV_{g}=0.1\omega _{0}$). (**a**) The singularity spectrum $f(\alpha )$ versus $\alpha$ is presented for different electron-phonon couplings $(\Lambda _{0})$. (**b**) The fractal dimensions $D_{q}$ versus *q* are shown for different electron-phonon couplings $(\Lambda _{0})$.

In molecular arrays and soft-hard interfaces, in general cases, the system is coupled to electron-phonon interactions. To study the spin transport in different nanodevices, it is crucial to consider the electron-phonon interaction. It is assumed that the vibrational mode interacts with a thermal reservoir of phonons with a strong enough coupling to maintain the thermal equilibrium of the phonon throughout the process. We have considered the multifractal properties of a $N=50~bp$ DNA chain with phonon energy $\omega _{0}=0.5~\textrm{eV}$ at $eV_{g}=0.1~\omega _{0}$, and $KT=0.1~\omega _{0}$ for various values of electron-phonon coupling $(\Lambda _{0})$ (Fig. 6a,b). The results show that the singularity spectrum $(f(\alpha ))$ becomes narrower when $\Lambda _{0}$ is higher than $\Lambda _{0}=0.1~\omega _{0}$ (Fig. 6a). This implies that a strong electron-phonon coupling enables phonon-assisted spin transport. The spin states become more delocalized when the electron-phonon coupling is higher than $\Lambda _{0}=0.1~\omega _{0}$. The fractal dimension of the system also decreases significantly at negative values of *q*, when $\Lambda _{0}$ increases (Fig. 6b). Therefore, a strong electron-phonon coupling leads to a phonon-assisted spin current and delocalized spin states in the system.

### The thermal effect and localization-delocalization transition

**Figure 7:**
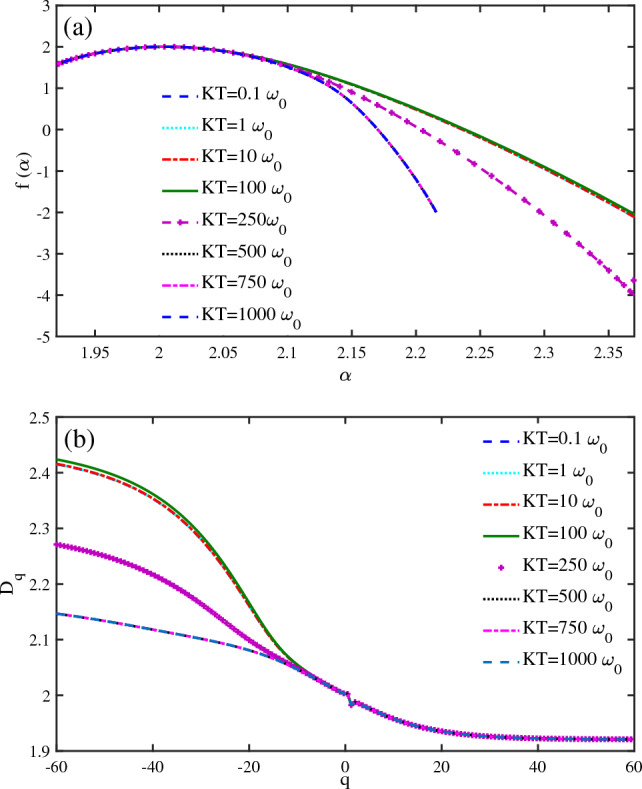
Multifractal analysis of spin states of a $N=50~bp$ DNA chain ($\omega _{0}=0.5~\textrm{eV}$, $\Lambda _{0}=0.1~\omega _{0}$, $eV_{g}=0.1\omega _{0}$). (**a**) The singularity spectrum $f(\alpha )$ versus $\alpha$ is presented for different phonon temperatures (*KT*). (**b**) The fractal dimensions $D_{q}$ versus *q* are shown for different phonon temperatures (*KT*).

The temperature dependence of the CISS effect demonstrates that temperature is a fundamental factor in spin transport in DNA. We have studied the temperature effect on the localization properties of spin states through multifractal analysis. The singularity spectrum divides the temperature ranges into intervals (Fig. 7a). The multifractality is the same for temperatures in the interval $KT\in [0.1-100]\omega _{0}$. The multifractality becomes weaker as the phonon temperature decreases. Therefore, the temperatures in $KT\ge 100~\omega _{0}$ up to $KT \approx 500~\omega _{0}$ have weaker multifractality than low temperatures. This trend continues until the temperatures in $KT\ge 500~\omega _{0}$ up to $KT \approx 1000~\omega _{0}$ have the weakest strength of multifractality. The weaker strength of multifractality means less spin state localization. So, the spin states become more delocalized as the phonon bath temperature increases. Previous experiments have shown that the spin polarization increases as temperature increases. We find that the spin states become more delocalized with increasing the temperature. If we assume these two results are related, we can infer that the more delocalized spin states lead to higher spin selectivity of the system. The $D_{q}$ diagram at different thermal ranges shows the same order for the fractal dimensions of the system (Fig. 7b). The fractal dimensions decrease as the temperature increases, which confirms the reduction in the strength of multifractality. The same results have been experimentally reported in previous studies.

### The effect of chain length on spin states

**Figure 8:**
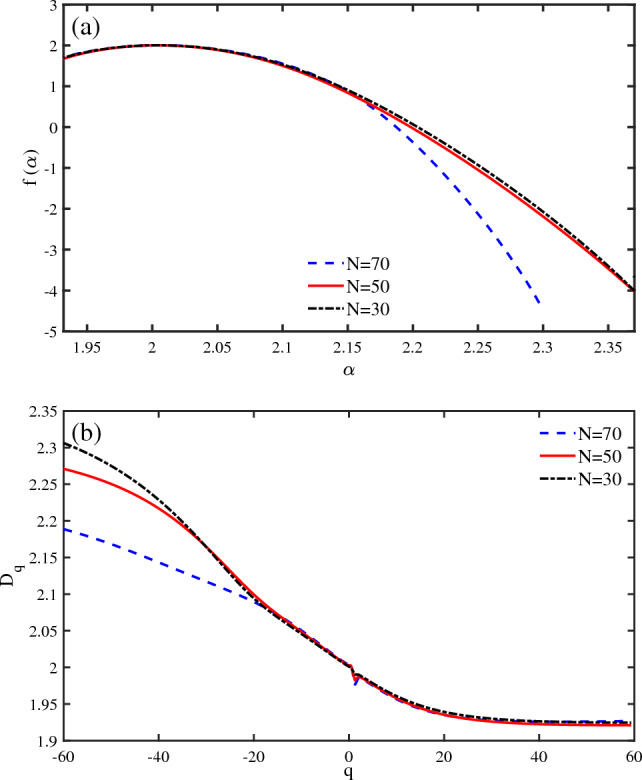
Multifractal analysis of spin states of a DNA chain ($\omega _{0}=0.5~\textrm{eV}$, $\Lambda _{0}=0.1~\omega _{0}$, $V_{g}=0.1\omega _{0}$, $KT=250~\omega _{0}$). (**a**) The singularity spectrum $f(\alpha )$ versus $\alpha$ is presented for different chain lengths (*N*). (**b**) The fractal dimensions $D_{q}$ versus *q* are shown for different chain lengths (*N*).

The charge transport and conductivity of a nanodevice is influenced by its length. Similarly, the spin selectivity of a chiral chain depends on the length of the system. In this regard, we have investigated the effect of molecular chain length on the localization-delocalization properties of DNA. The singularity spectrum shows a decrease in the strength of multifractality in longer chains (Fig. 8a). Thus, the spin states are more extended in longer DNA sequences as spin polarization is higher for longer chiral molecular chains. A similar result is obtained from the $D_{q}$ diagram (Fig. 8b). The fractal dimensions decrease with increasing the chain length. Therefore, the spin states are less localized for longer sequences, as is verified in spin transport experiments.

## Discussion

Spin filtering, the selective transmission of electrons with different spin orientations, has been observed in various chiral molecules and their aggregates, such as DNA, amino acids, oligopeptides, and helicenes. There is a large variety of experimental evidence of spin-selective transport in chiral organic molecules^40–42^. In the chirality-induced spin selectivity (CISS) experiments, the nonmagnetic molecule respects time-reversal symmetry. Different approaches have proposed a model to study spin transport in chiral chains, such as DNA, that assumes an electron moving in a double helical path under the effect of a helical electrostatic potential^25,43,44^. To achieve high spin selectivity, it is required a mechanism that disrupts the electronic phase by interacting with virtual Büttiker leads. When an electron is transmitted through the molecular system, it may experience inelastic scattering events, which lead to the loss of phase memory. This can be simulated by attaching each site of the molecule to a Büttiker’s virtual electrode. Büttiker’s virtual probes method is a helpful tool for studying the dephasing effect in a quantum system. Now, it is widely employed in topological insulators and spin systems^45–47^. Büttiker’s virtual electrode is similar to the real one, because their Hamiltonians are identical to each other. However, with Büttiker’s probes, zero current flow must be enforced through them in order to have current conservation, because the probes are not necessarily physical terminals but mathematical artifacts to induce dephasing^48^. Therefore, a small dephasing is necessary for the existence of the spin polarization. Indeed, it is reasonable to assume a small dephasing because the dephasing occurs inevitably in the experiment. In fact, the dephasing has two effects: (i) it promotes the openness of the two-terminal device and generates spin polarization^49^; (ii) it makes the charge loses its phase and spin memory. On the one hand, the dephasing improves the openness of the molecule by coupling each site to a Büttiker virtual lead, which gives rise to the spin selectivity effect^49^. This role of the dephasing is dominant when it is not quite strong, and then polarization increases with dephasing strength at first. On the other hand, the dephasing, which is introduced to simulate the inelastic-scattering events, leads to the loss of the electron phase memory and subsequently suppresses the spin selectivity effect. The second role of the dephasing competes with the first one and becomes dominant in the strong dephasing regime. In this situation, spin selectivity slowly declines by further increasing dephasing strength. As a result, the dependence of spin polarization on dephasing strength is not monotonic. However, additional factors besides spin-orbit coupling are needed for spin-dependent electrical properties. To obtain spin-dependent electrical properties, one must artificially violate time reversal symmetry^50^, two energy levels per site, and have different electronic-coupling elements between the channels^51^. The electron is modeled by a wave packet, and the criteria for the emergence of a persistent spin-current and the desirable spin-splitting are determined. We focus on the case where there is spin-transfer without charge transport. It is demonstrated that spin-orbit coupling does not lead to an unbalanced spin-polarized current, but it induces a spatial separation between spin-up and spin-down components. The spin-orbit coupling makes the opposite spin polarization move with opposite velocities along the molecule, resulting in a finite spin-current. This spatial separation of the spin components implies that helicoidal molecules can be used as spin filters in spintronic devices.

Moreover, the DNA structure can change and create the phonon, affecting charge transmission. Recent theoretical studies have shown that spin polarization can be enhanced by polarons^10^, electron correlation^52^, and phonons^53^. The transport properties of soft biological systems can be significantly influenced by their vibrational modes, which are abundant at room temperature^54^. Therefore, charge transfer often occurs via polarons^55–57^. The environment is polarized by the polaron motion along the molecule, which leads to complex charge dynamics. Each polaron has a phonon cloud that makes its mass much larger than the electronic one. The polaron band becomes narrower due to phonons, but the SOC does not change. Therefore, the energy window showing CISS covers a more significant fraction of band energies. It was found that the polaron spin is polarized as it moves through the chiral system^10,58^. Moreover, a significant asymmetry in the magnetoresistance due to a spin-dependent interaction between electrons and chiral phonons is reported^13^. These phonon modes, common in chiral molecules, depend on the geometrical structure of the system like the ordinary acoustic modes. However, most of the vibrational modes in molecules are localized. It was demonstrated that localized modes also enable the CISS effect^13^. When the polarons move along the molecule, they create local distortions in the surrounding vibrations by emitting and absorbing phonons. This enables them to access various levels in the energy space, including the region of states that show strong spin selectivity. Besides polarizing the current, they also undergo large fluctuations as they move along the molecule, which results in nonlinear current-voltage relations. We have studied CISS in the presence of electron-phonon interactions. In the strong electron-phonon regime, the polarons carry both the charge and the spin, which cause significant polarization of the environment. Polaron fluctuations lead to clear evidence of CISS in spin-dependent measurements. The critical point is that the molecule’s chiral structure only affects the (unperturbed) electronic spectrum, while the phonons can be simple like the optical modes are studied. We have observed a phonon-assisted spin state delocalization, which leads to a phonon-assisted spin current. The phonon-assisted spin current was generated in single molecular systems, previously^59^. Chiral phonons are a novel phenomenon that can carry angular momentum, which can be added as a magnetic contribution to the total moment. Chiral phonons can create a nontrivial spin texture in a nonmagnetic electronic structure. By modeling a system with electron-phonon interaction, it is shown that chiral phonons can transfer their angular momentum to the electrons, which become spin-polarized as a result^60^. An electric field makes the site energies of the helix aperiodic as in the Aunry-André-Harper (AAH) form, which breaks the symmetry among up and down spin currents. Under this condition, a finite bias drop along the helix yields different spin-specific junction currents in two bias polarities, resulting in a spin current rectification^61^. In the absence of either helicity or electric field, no such behavior is observed. Following the pioneering work of Aviram and Ratner^62^, a considerable amount of work, theoretically^63,64^ and experimentally^65,66^, has been made on how to get charge current rectification at the molecular scale level^67–69^. However, the studies related to spin current rectification^70^ (SCR) are relatively scarce. The fundamental requirements for SCR are: (i) finite mismatch between up and down spin energy channels and (ii) asymmetric transmission line shapes in two bias polarities. Breaking the symmetry, we can have a finite mismatch among the two different spin-dependent energy channels, and thus, we can get spin-specific electron transport. In a helix system, we can do it by applying an electric field perpendicular to the helix axis. In presence of this field, the system becomes a correlated disordered one, analogous to the well-known Aubry-André-Harper form^71,72^, and changing the field strength and its direction, we can selectively monitor the transport behavior. When a finite bias is applied across the helix, an additional field dependence occurs, which further modifies the site energies of the helix. Depending on the potential drop, the site energies get modified, and together with the correlated disorder, we get different transmission spectra for positive and negative bias polarities, which results in a finite spin current rectification. No such phenomenon can be observed in the absence of helicity or external electric field. Therefore, the spin transfer can be justified by a gate voltage indirectly^34,73^. The spin-orbit interaction and an external electric field can create additional overlap pathways between the electron-bearing orbitals because they couple intra-atomic orbitals^74,75^. These pathways can control the spin transport properties of the system^76^. Moreover, the experiments demonstrated that time-reversal symmetry is broken by an external voltage^77^. So, the applied voltage (breaks time-reversal symmetry) causes spin selectivity due to the coupling of the direction of electron motion with the spin orientation. Thus, the bias direction selects an electron spin polarization^78^.

The magnitude of spin polarization is higher for longer chiral molecular chains because they experience more spin-selective scattering from chiral potentials in such systems^79, 80,10^. It was established that for a certain group of oligomers, like peptides or DNA, the spin polarization changes nearly linearly to the length of the oligomer for the initial few nanometers^26,40,81^. A pioneer experimental work was presented the length-dependent spin polarization in dsDNA^40^. They showed that increasing the length of the dsDNA tends to increase the absolute value of electron spin polarization. Also, the experimental data based on the results of spin selective transport obtained with magnetic conducting atomic force microscopy (mc-AFM) present the length-dependence of the spin current measured for dsDNA^82^. The spin polarization depends linearly on the length. The favored spin is transmitted more efficiently as a length function than the unfavored spin^83^. These results support the notion that due to the coupling between the electron’s linear momentum and its spin, the backscattering of the favored spin is reduced. A possible way to achieve the large CISS is an interplay between SOC and electron-phonon coupling^20,53,84^. CISS increases with temperature due to electron-phonon coupling. It is proposed that a mechanism involving spin-orbit and electron-phonon coupling in CISS leads to nonequilibrium spin accumulation on the molecule^85^. The experimental results investigated that the CISS is enhanced with increasing temperature^85^. The manifestation of the CISS effect in scattering experiments can be explained by the emergence of chirality-dependent correlations between the electron spin and its direction of propagation. The SOC of organic structures, which is on the order of a few meV, is much smaller than the coupling between neighboring sites. Consequently, the downturn of the polarization occurs well above room temperature^10^. Recently, it has been shown that the CISS effect possesses unexpected dependencies on the temperature. Experiments performed on such organic compounds indicate that such magnetic properties are not only strong and stable at room temperature, but also the same properties dramatically wane as the temperature drops toward 0 K. In other words, these compounds become magnetically stabilized, and even reinforced, with increasing temperature, which is quite the opposite of the predictions that can be made using the conventional theory for magnetism^86^. Therefore, our theoretical results can be used to explain the related experimental results. Vibrations can play a crucial role in explaining the phenomenon, as pointed out by Fransson^13,52^. It is proposed to exploit CISS to lock spin, and charge. This CISS-mediated spin-charge locking does not rely on the small Zeeman energy scale^87^ and, it therefore could work at much higher temperatures than those employed in semiconductor quantum dots or impurity spins^88^. Although the coherence time is not the current limiting factor, it could be further enhanced, by controlling phonon-mediated relaxation and dephasing processes (which dominate at relatively high temperatures). This can be done through a careful design of the molecular structure (coordination geometry, ligand rigidity), electronic gaps, and spin-phonon couplings, as suggested previously^23,89–92^. An essential point for quantum technology applications is related to the temperature dependence of the CISS effect and to the possibility of tuning it by engineering the molecular structure. Indeed, recent results suggest that CISS efficiency increases with temperature, whereas coherence times of molecular qubits typically decrease. However, coherent manipulations of molecular qubits had been performed even at room temperature. This temperature resilience could be further increased by engineering ligands and removing neighboring nuclear spins. In the hopping regime, carriers get more delocalized as temperature increases due to the interaction between electrons and phonons^93,94^. In this regime, resistance decreases as the rate of electron-phonon scattering increases^93^. In the transport phenomena, the electron-phonon coupling, which leads to thermal and decoherence effects in transport, is constrained by symmetry in the presence of spin polarization. We have used multifractal analysis to investigate the localization-delocalization transition in spin states of DNA chains.^95^. Multifractal analysis can characterize the critical behavior in biomolecules^96^. Anderson localization, a theory that describes how waves are scattered and trapped in disordered media, has been applied to various fields of physics, such as condensed matter physics^97^, chaos theory^98^, and photonics^99^. It is demonstrated from the multifractal analysis that the spin states are extended when the singularity spectrum is narrow. In this condition, the fractal dimensions get lower values. The obtained result for localized/delocalized spin states concerning the applied voltage, thermal effect and, length of polymer confirms the discussed matter.

## Conclusion

The manuscript proposed a novel and intriguing spintronic nanodevice based on a flexible bio-material that originating DNA chains. The sensitivity, selectivity, and challenges of localization of spin states are discussed. We have theoretically studied the spin transport properties using multifractal analysis. The results show a nearly pure spin current in DNA chains and a rectified spin behavior concerning a gate voltage. The results demonstrated the control parameters as a critical tool for modulating the spin state localization/delocalization. Using the external stimuli, we can justify the transition regions in our spin device. The controlled transmission of spins through organic DNA molecules has potential applications in molecular and molecular-nano hybrid spintronics and sensors.

## Methods

### Multifractal analysis

Energy eigenstates are crucial components in quantum mechanical systems^100^. Statistical analysis of the eigenstate coefficient is a suitable method to identify the critical behaviors of the transitions between localized and delocalized states in disordered systems^101^. The distribution of eigenstates was investigated directly for quantum maps^102^, many-body systems^103,104^, quantum billiards^105,106^, and random-matrix ensembles^107,108^.

The fluctuations in eigenfunctions can be described by a set of multifractal dimensions $D_q$ that depend on the scaling of the inverse mean eigenfunction participation numbers with the system size *N*^95^:

$$\begin{aligned} \left\langle \sum _{i=1}^{N} |\psi _{i}|^{2q}\right\rangle \sim N^{-(q-1)D_{q}} \end{aligned}$$

where *q* is a parameter, and $\langle ... \rangle$ is the average over some eigenstates within an eigenvalue window and over the ensemble. It is known that the multifractal phenomenon occurs in a quantum system. The multifractality of quantum states indicates the statistical properties of states. It plays an essential role in studying the phase transitions of various quantum systems^109,110^. To examine the localization/delocalization regimes, one can apply multifractal analysis of eigenstates. It is confirmed that the wave functions of the system in the localization-delocalization transition exhibit multifractal fluctuations^111^. The fractal dimension $D_{q}$ can be defined through the mass exponent $\tau _{q}$ as $\tau _{q}=D_{q}(q-1)$. In Anderson localized phase, since each eigenstate is confined to a finite number of sites, the fractal dimension is zero ($D_{q}=0$). On the other hand, ergodic quantum eigenstates are those states for which at least a finite fraction of the coefficients in the given basis have an enormous contribution, and thus, $D_{q}=d$, where *d* is the topological dimension of the system.

The singularity spectrum $f(\alpha )$ is written via the Legendre transformation as follows^101^:

14 $$\begin{aligned} f(\alpha )=\alpha q-\tau _{q}. \end{aligned}$$

Based on the definition of the singularity spectrum $f(\alpha )$, it is clear that multifractal time series are characterized by a broad $f(\alpha )$, while monofractal ones have a narrow $f(\alpha )$. In other words, the strength of the multifractality can be seen as the width of $f(\alpha )$, $\Delta \alpha =\alpha _{max}-\alpha _{min}$, such that as $\Delta \alpha \rightarrow 0$, we have a loss of multifractality. The singularity spectrum can measure the degree of electron state localization. The singularity diagram of the system shrinks to be narrow when the electron states become more extended.

## Data Availability

The datasets used and/or analyzed during the current study are available from the corresponding author upon reasonable request.
